# Novel Tacrine-Scutellarin Hybrids as Multipotent Anti-Alzheimer’s Agents: Design, Synthesis and Biological Evaluation

**DOI:** 10.3390/molecules22061006

**Published:** 2017-06-16

**Authors:** Katarina Spilovska, Jan Korabecny, Vendula Sepsova, Daniel Jun, Martina Hrabinova, Petr Jost, Lubica Muckova, Ondrej Soukup, Jana Janockova, Tomas Kucera, Rafael Dolezal, Eva Mezeiova, Daniel Kaping, Kamil Kuca

**Affiliations:** 1 Department of Toxicology and Military Pharmacy, Faculty of Military Health Sciences, Trebesska 1575, 500 01 Hradec Kralove, Czech Republic; k.spilovska@gmail.com (K.S.); korabecny.jan@gmail.com (J.K.); vsepsova@gmail.com (V.S.); daniel.jun@unob.cz (D.J.); martina.hrabinova@unob.cz (M.H.); petr.jost@unob.cz (P.J.); lubica.muckova@unob.cz (L.M.); Tomas.kucera2@unob.cz (T.K.); 2 National Institute of Mental Health, Topolova 748, 250 67 Klecany, Czech Republic; ondrej.soukup@fnhk.cz (O.S.); eva.mezeiova@gmail.com (E.M.); Daniel.Kaping@nudz.cz (D.K.); 3 Biomedical Research Centre, University Hospital Hradec Kralove, Sokolska 581, 500 05 Hradec Kralove, Czech Republic; jana.janockova@gmail.com (J.J.); rafael.dolezal@centrum.cz (R.D.)

**Keywords:** Alzheimer’s disease, 6-chlorotacrine, scutellarin, enzyme inhibitor, acetylcholinesterase, butyrylcholinesterase

## Abstract

A novel series of 6-chlorotacrine-scutellarin hybrids was designed, synthesized and the biological activity as potential anti-Alzheimer’s agents was assessed. Their inhibitory activity towards human acetylcholinesterase (*h*AChE) and human butyrylcholinesterase (*h*BChE), antioxidant activity, ability to cross the blood-brain barrier (BBB) and hepatotoxic profile were evaluated in vitro. Among these compounds, hybrid **K1383**, bearing two methylene tether between two basic scaffolds, was found to be very potent *h*AChE inhibitor (IC_50_ = 1.63 nM). Unfortunately, none of the hybrids displayed any antioxidant activity (EC_50_ ≥ 500 μM). Preliminary data also suggests a comparable hepatotoxic profile with 6-Cl-THA (established on a HepG2 cell line). Kinetic studies performed on *h*AChE with the most active compound in the study, **K1383**, pointed out to a mixed, non-competitive enzyme inhibition. These findings were further corroborated by docking studies.

## 1. Introduction

Alzheimer’s disease (AD) is a progressive neurodegenerative brain disorder characterized by gradual degeneration and loss of neurons [1]. According to the World Alzheimer Report 2015, approximately 46.8 million people suffer from AD and other forms of dementia worldwide. This number is expected to increase due to the rapid growth of the aging population, reaching 74.7 million by 2030 and 131.5 million by 2050 [2]. To date the pathogenesis of this neurodegenerative disease has not been fully elucidated. However, several factors have been linked to play an important role in AD pathogenesis, including loss of cholinergic transmission, excessive accumulation of Aβ aggregates extracellularly in the brain, accumulation of tau protein intracellularly, oxidative stress with free radicals formation, biometal dyshomeostasis, glutamate excitotoxicity, inflammatory processes as well as disturbed mitochondrial transport to synaptic terminals [3,4].

Current AD therapeutic approaches rely on the administration of cholinesterase inhibitors (ChEIs) (donepezil, galantamine, and rivastigmine) and a non-competitive *N*-methyl-d-aspartate receptor antagonist (memantine), which contribute to an improvement of cognitive functions, but do not prevent from progressive neurodegeneration [5,6].

The multifactorial character of AD requires new therapeutics to successfully affect multiple AD-relevant pathways responsible for the pathogenesis of the disease [7]. The so-called multi-target directed ligands (MTDLs) strategy has been postulated as a promising one [8,9,10,11,12,13,14,15,16]. Accordingly, the most widely accepted theory for drug discovery in AD by medicinal chemists is tied to the modification of acetylcholinesterase inhibitors (AChEIs). The possible benefit of MTDLs are reduced incidence of possible drug-drug interactions, a higher efficacy of these drugs and concomitantly a simple dosing mode which may enhance patient compliance [17]. However, today it remains unclear which pathological aspects of AD are best to target to resolve the multiple nature of the disease.

6-Chlorotacrine (6-Cl-THA) is a potent acetylcholinesterase inhibitor (AChEI) widely used as a basic moiety to be the introduced into novel MTDLs. It displays toxicity comparable to that of tacrine (THA), [18] but higher AChE inhibitory activity. Several 6-Cl-THA-based hybrids, such as 6-Cl-THA-melatonin hybrids [19,20], 6-Cl-THA-1,4-naphthoquinone hybrids [21], 6-Cl-THA-caffeic acid heterodimers [22], 6-Cl-THA-Trolox heterodimers [23], hybrids combining 6-Cl-THA with caffeic, ferulic and lipoic acid [24] and 6-Cl-THA-ferulic acid heterodimers [25], were synthesized and evaluated in vitro, revealing interesting biological profiles.

**Figure 1:**
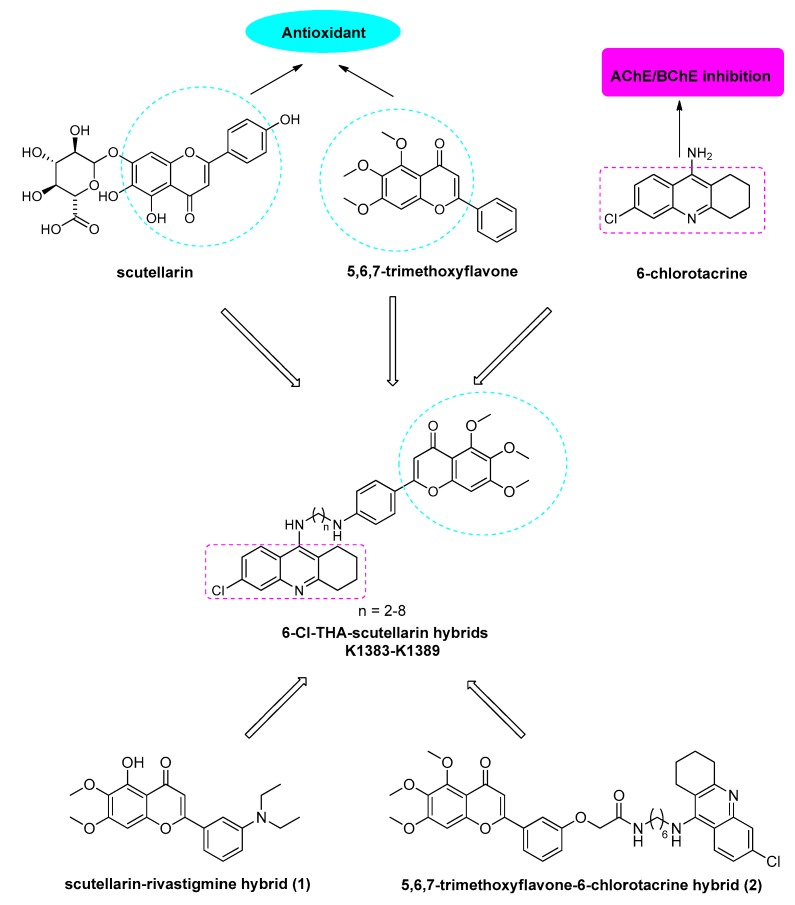
Design strategy for the 6-Cl-THA-scutellarin hybrids **K1383**–**K1389**.

Scutellarin (4′,5,6-trihydroxyflavone-7-glucuronide, Figure 1) is a flavone firstly isolated from the Chinese herb *Erigeron breviscapus*. Its wide spectrum of pharmacological properties include free radical scavenging ability, anti-inflammatory efficacy, neuroprotection and capability to inhibit Aβ fibril formation [26,27,28]. Unfortunately, due to its poor solubility, weak oral absorption and poor ability to cross the blood-brain barrier (BBB), the clinical relevance of scutellarin is limited [29,30]. Recently, Sang et al. synthesized and biologically evaluated scutellarin-rivastigmine hybrids as multifunctional agents for AD treatment. The most prominent of them was compound **1** (Figure 1) with good anticholinergic, antioxidant, biometal chelating and neuroprotective properties. Moreover, **1** was able to cross the BBB [31].

5,6,7-Trimethoxyflavone (Figure 1) is a compound structurally similar to scutellarin exerting a broad biological profile, ranging from free radical scavenging ability, the inhibition of Aβ fibril formation, anti-cancer properties and anti-inflammatory activity including neuroprotection [32,33,34]. Moreover, there is a wide spectrum of flavones and natural extracts with potential antioxidant and neuroprotective activity [35,36,37,38,39,40,41]. Liao’s group recently reported the development of novel 5,6,7-trimethoxyflavone-6-Cl-THA hybrids as potential anti-AD agents. The most potent hybrid **2** (Figure 1) exhibited good AChE inhibitory activity, potential antioxidant and neuroprotective activity, ability to cross the BBB and significant inhibitory potency of self-induced Aβ aggregation [42].

Based upon the aforementioned findings, we report the design, synthesis and biological evaluation of a novel series of 6-Cl-THA-scutellarin hybrids **K1383**–**K1389** (Figure 1), including their AChE and BChE inhibitory activity, the antioxidant activity, molecular modeling studies, ability to cross BBB, as well as the evaluation of their hepatotoxicity on HepG2 cell line.

## 2. Results and Discussion

### 2.1. Chemistry

**Scheme 1:**
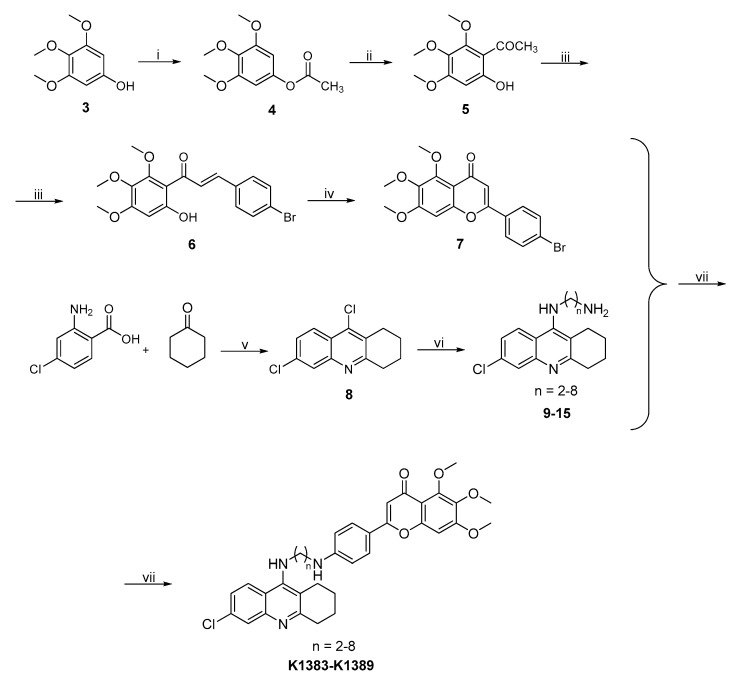
Synthetic procedure for 6-chlorotacrine-scutellarin hybrids **K1383**–**K1389**. *Reagents and conditions*: (i) sodium acetate, acetic anhydride, 110 °C; (ii) BF_3·_Et_2_O, acetic acid, 70 °C; (iii) 4-bromo-benzaldehyde, EtOH, 30% KOH, r.t.; (iv) I_2_, DMSO, 170 °C; (v) POCl_3_, 130°C; (vi) phenol, appropriate 1,ω-diamines, 130 °C; (vii) Pd_2_(dba)_3_ + BINAP + NaOtBu, dry toluene, 110 °C.

The general synthetic procedure for 6-Cl-THA-scutellarin hybrids is outlined in Scheme 1. *O*-Acetylation of the starting compound 3,4,5-trimethoxyphenol (**3**) followed by Fries rearrangement using BF_3_·Et_2_O as catalyst were used to prepare intermediate the 1-(6-hydroxy-2,3,4-trimethoxy-phenyl)ethane-1-one (**5**) in good yield (76%) [43]. Then, **5** reacted with 4-bromobenzaldehyde in the presence of 30% aqueous solution of KOH to obtain chalcone intermediate **6** as the *trans* isomer, the same geometric configuration of the compound found in the literature [44]. This was also supported by the corresponding ^1^H-NMR spectrum of **6** showing coupling constants of the vicinal hydrogens of the double bond in the range of 12–18 Hz [45]. Cyclization of **6** with iodine in dimethylsulfoxide (DMSO) yielded scutellarin synthon **7** in a relatively low yield (20%) [44]. 6,9-Dichloro-1,2,3,4-tetrahydroacridine (**8**) was prepared as a brown solid in good yield (70%) from 4-chloroanthranic acid with cyclohexanone in the presence of POCl_3_ [46]. The spectral data were in good agreement with the literature characterization [46]. The treatment of **8** with appropriate 1,ω-diamines in the presence of phenol afforded the required *N*-(6-chloro-1,2,3,4-tetrahydroacridin-9-yl)alkane-1,ω-diamine intermediates **9**–**15** (70–95%) [23].

The synthon **8** and the 1,ω-diaminotacrines **9**–**15** were utilized for formation of the desired 6-Cl-THA-scutellarin hybrids **K1383**–**K1389** in a Buchwald-Hartwig cross-coupling reaction under catalysis of Pd_2_(dba)_3_ + BINAP + NaOtBu in dry toluene [47]. The final compounds were obtained as yellow powders in yields ranging from 15–88%. Structural determination and signal assignments were accomplished by the application of the usual combination of ^1^H- and ^13^C-NMR spectroscopy. Final compounds were also characterized by high-resolution mass spectrometry (HRMS) and melting points.

### 2.2. Biological Evaluation

#### 2.2.1. Cholinesterase Inhibition 

The in vitro inhibitory activities of the seven novel 6-Cl-THA-scutellarin hybrids **K1383**–**K1389** were determined on the models of recombinant *h*AChE (E.C. 3.1.1.7) and plasmatic *h*BChE (E.C. 3.1.1.8) using spectroscopic Ellman’s method [48,49] and compared with 6-Cl-THA as reference (Table 1). All new heterodimers were found to be potent inhibitors of both cholinesterases. In general, these hybrids exerted high affinity towards *h*AChE with IC_50_ values ranging from 1.63 nM for the most active hybrid **K1383** to the least potent **K1387** 31.0 nM. Structure-activity relationship (SAR) related to *h*AChE revealed that derivatives bearing shorter linkers slightly exceeded the activity of those with longer chains. Especially derivatives **K1383**, **K1384** and **K1385** displayed single nanomolar digit *h*AChE inhibition, surpassing the inhibition capability of 6-Cl-THA by one order of magnitude. Regarding *h*BChE inhibition, all the hybrids were less potent inhibitors of this enzyme, with IC_50_ values lying in the micromolar to submicromolar region. The highest inhibition power for *h*BChE was demonstrated by hybrid **K1384** (IC_50_ = 174 nM). It is well established that the activity of AChE decreases during the course of AD, while that of BChE increases. Several reports indicate the ability of BChE to compensate the lack of AChE thus continuing in regulation of cholinergic transmission. Therefore, the agents with affinity to both cholinesterases could play a more profound role in the AD therapy [50]. The selectivity index (SI), reflecting the relative preference for *h*AChE over *h*BChE, was calculated presuming high selectivity of novel 6-Cl-THA-scutellarin family for *h*AChE. From this point of view, **K1385**, showing the highest SI for *h*AChE (SI = 1687) which is comparable to that for donepezil (SI value for donepezil = 1100) [51], can be highlighted. Previously prepared compound **1** [31] showed sub-micromolar and micromolar value inhibitory activity toward rat AChE and rat BChE, respectively. Hybrid **2** [42] exhibited sub-micromolar inhibitory activity toward *Ee*AChE and *h*AChE and micromolar *eq*BChE inhibitory activity. Although these compounds were tested on different type of enzyme than novel 6-Cl-THA-scutellarin hybrids, they displayed in general better inhibitory potency against *h*AChE.

#### 2.2.2. Kinetic Analysis

**Figure 2:**
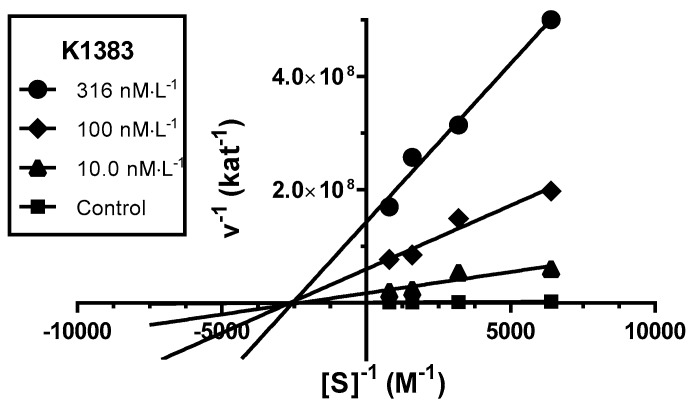
Steady-state inhibition of AChE hydrolysis of substrate acetylthiocholine (ATCh) by compound **K1383** at different concentrations. Lineweaver-Burk plots of initial velocity at increasing substrate concentrations (0.156–1.250 mM) are presented. Lines were derived from a linear regression of the data points.

To determine the interaction of **K1383** with *h*AChE, a kinetic study was carried out. Enzyme rate curves were obtained at several substrate concentrations of the inhibitor. The type of inhibition was determined from the nonlinear regression analysis, which confirmed mixed, non-competitive mode of AChE inhibition (*p* < 0.05), consistent with graphical representation of corresponding Lineweaver-Burk plot in Figure 2, where all of the lines intersect on the one point of the *x*-axis.

Inhibitors bind reversibly to both free AChE and AChE-substrate complex with equal affinity (*K*_i_ = *K*_i_′) thus affecting the binding of substrate in the active site of enzyme and interacting with its allosteric peripheral anionic site (PAS). Interaction in this site causes conformational changes of the enzyme, including its active site. *K*_m_ remained unchanged, while *V*_max_ was reduced at higher concentration of **K1383**. The corresponding *K*_i_ value of 2.75 ± 0.32 nM was calculated from measured data of the tested inhibitor.

#### 2.2.3. Antioxidant Activity 

To determine the antioxidant activity of novel 6-Cl-THA-scutellarin hybrids, we used the 1,1-diphenyl-2-picrylhydrazyl (DPPH) assay [52] with Trolox as positive control and 6-Cl-THA, **1** and **2** as standards. As shown in Table 2, the results suggest that all selected hybrids exhibited weak antioxidant activity (EC_50_ higher than 10^−4^ M) compared to Trolox, **1** and **2**. The free phenolic group of **1** is presumably able to easily undergo keto-enol tautomerism which has previously been postulated to be responsible for radical scavenging [53]. Within the family of 6-Cl-THA-scutellarin hybrids, all the phenolic hydroxyls of scutellarin moiety are hindered by methyl containing methoxy groups and thus unable to exert such an effect under in vitro conditions. However, the antioxidant activity of **K1383**–**K1389** in vivo may occur due to metabolism of the compound whereby demethylation of one or more methoxy groups can take place. This is known to be the case of 7-methoxytacrine and its major metabolite found in vivo, 7-hydroxytacrine [54].

#### 2.2.4. Hepatotoxicity

In consequence of the hepatotoxicity of tacrine and some of its derivatives a 3-(4,5-dimethylthiazol-2-yl)-2,5-diphenyltetrazolium bromide (MTT) assay [56] was carried out to determine the potential hepatotoxicity of novel 6-Cl-THA-scutellarin hybrids **K1383**–**K1389 [57]**. Cytotoxic effects were assayed on human liver carcinoma (HepG2) cell lines and the results are displayed in Table 3. After 24 h of incubation, only two novel derivatives, namely **K1388** and **K1389**, bearing seven and eight methylene linkers, showed slightly suppressed cytotoxicity compared to the reference compound 6-Cl-THA. These results should however, be taken with some precaution due to the limited solubility and unknown pharmacological profile of **K1383**–**K1389** in vivo (ADME properties) [23].

#### 2.2.5. In Vitro Blood-Brain Barrier Permeation Assay

The ability of novel derivatives from 6-Cl-THA-scutellarin subset to permeate the BBB was predicted using a parallel artificial membrane permeation assay of the BBB (PAMPA-BBB) described by Di et al. [58,59]. All the data are gathered in Table 4. Based on the obtained results, three of the tested hybrids (compounds **K1384**, **K1388**, **K1389**) displayed the potential to cross the BBB, whereas the rest of the series demonstrated uncertain CNS permeation in the passive diffusion model.

#### 2.2.6. Molecular Docking

Initially, it should be noted that both cholinesterases share high degrees of homology (50–60%) [60]. However, such homology is not enough to provide same degree of ligand affinity to the active site of both AChE and BChE. The BChE active site is much bulkier compared to that of AChE. This results from some the absence of aromatic residues within the active gorge of BChE and also observable structural differences near the catalytic anionic site (CAS) of BChE [61]. In addition, broadly cited PAS containing several aromatic residues and located at the rim of the cavity gorge is highly conserved and specific for AChE, whereas in PAS in the BChE active site the number of such residues is greatly reduced [62]. This is also the reason for the different inhibition efficacies of the compounds under the study, i.e., the 6-Cl-THA-scutellarin family.

Having all these prerequisites in mind, we have conducted molecular modeling studies with **K1383**, **K1384** and **K1385** in order to explore their high affinity towards *h*AChE or *h*BChE and high *h*AChE selectivity profile observed mainly in case of **K1385**. The selection of *h*AChE and *h*BChE for the computational studies is rationally in accordance with in vitro studies where the enzymes of human origin were also exploited. Firstly, we have taken *h*AChE (PDB ID: 4EY7) and *h*BChE (PDB ID: 4BDS) from the Protein Data Bank as source receptors for embedding the ligands and, ultimately, explore their binding modes via flexible ligand docking protocol [63,64].

**Figure 3:**
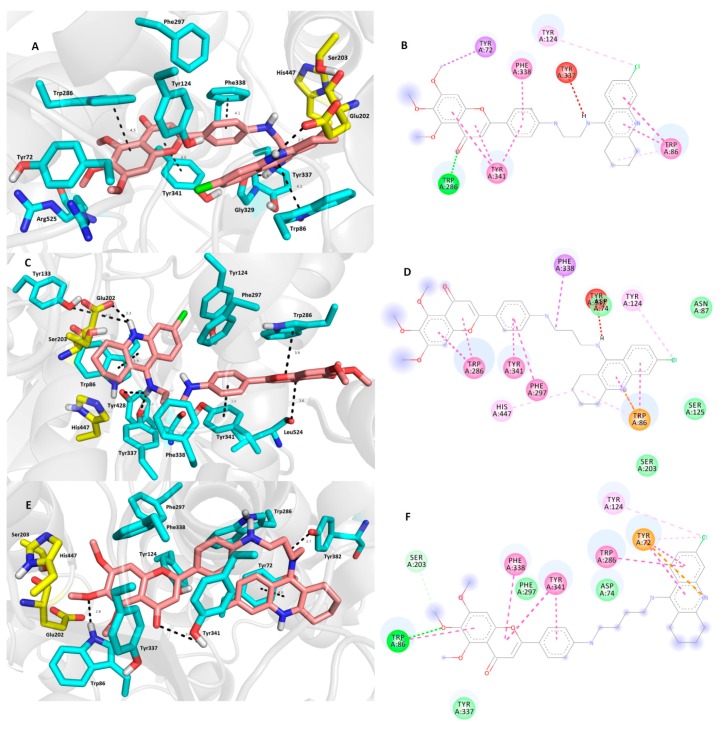
Superimposition of **K1383** (**A**,**B**), **K1384** (**C**,**D**) and **K1385** (**E**,**F**) in *h*AChE active site. Important amino acid residues involved in enzyme-ligand interaction are depicted in light-blue carbon atoms, ligands in salmon carbons and catalytic triad in yellow (**A**,**C**,**E**). Three-dimensional figures (**A**,**C**,**E**) were created by PyMol viewer 1.3, 2D figures (**B**,**D**,**F**) were built by BIOVIA Discovery Studio software.

Top-scored docking poses of **K1383**, **K1384** and **K1385** in the *h*AChE active site are illustrated in Figure 3. Focused on **K1383** (−13.3 kcal/mol; Figure 3A,B), the ligand revealed a dual binding site pattern of inhibition with the scutellarin moiety protruding out of the cavity gorge and THA moiety stacked in the CAS of the enzyme. Such an orientation of THA scaffold is highly consistent with other recently described THA dual binders like tacrine-tianeptine or 7-methoxytacrine-adamantylamine heterodimers [65,66,67]. On contrary, it has to be stated that opposite orientation, i.e., THA lodging within PAS region, can be also found in the literature like in 7-methoxytacrine-*p*-anisidine, tacrine-Trolox or 7-methoxytacrine-donepezil like compounds [68,69,70]. In case of **K1383**, the 6-Cl-THA moiety is facing Trp86 (4.1 Å) in a π-π/cation-π parallel manner. The secondary amino group of 6-Cl-THA is further stabilized by a hydrogen bond to Gly329 (1.4 Å). Glu202 (2.5 Å) is the only amino acid residue from the catalytic triad being involved in ligand enzyme interactions, forming a hydrogen bond with a protonated nitrogen from the 6-Cl-THA template. The high enzyme affinity of **K1383** is caused also by proper orientation of the chlorine atom to Tyr124. Scutellarin from **K1383** is sandwiched between Trp286 (4.5 Å), a key aromatic residue of PAS, and Tyr341 (4.0 Å) by slightly deflected parallel π-π stacking. Moreover, some other hydrogen bond interactions to scutellarin can be also found with Arg525. The scutellarin-attached phenyl ring provided a π-π interaction in the mid-gorge of *h*AChE to Phe338. Many other hydrophobic interactions contribute to **K1383**-*h*AChE complex constriction (e.g., with Tyr124, Tyr337 or Phe297). On the other hand, Tyr337 seems to form unfavorable hydrogen bonds to the secondary amino group of 6-Cl-THA.

Very close spatial orientation to that observed between **K1383** and *h*AChE can be found within the *h*AChE-**K1384** complex (−13.6 kcal/mol; Figure 3C,D). More in detail, the 6-Cl-THA scaffold is proximally lodged while the scutellarin moiety is situated distally at the cavity entrance. The THA core is stacked by π-π/cation-π interactions against Trp86 (3.9 Å). Interestingly, more hydrogen contacts can be found compared to **K1383**-*h*AChE, like between the secondary amino group of 6-Cl-THA moiety and Tyr337 (2.4 Å) and Tyr428 (2.5 Å). However, Tyr341 is involved in an unfavorable hydrogen bond to the amino group of the THA moiety. Additionally, the charged nitrogen from THA supports ligand anchoring via hydrogen bonding to Glu202 (2.3 Å). Other catalytic triad residues are not involved in enzyme-ligand interactions. Contrary to the *h*AChE-**K1383** complex, Tyr341 (3.8 Å) provided a parallel—interaction to the scutellarin-attached phenyl ring. This is presumably caused by the one methylene group longer tether and a more favorable ligand arrangement in the gorge. The scutellarin moiety faces directly to Trp286 (3.9 Å) forming a parallel π-π interaction. In general, slightly suppressed potency can be attributed to the missing interaction of the scutellarin moiety with Tyr341. Overall binding poses of **K1383** and **K1384** within *h*AChE active site are in good agreement with the docking results obtained for **2**-*Tc*AChE complex [71].

Opposite positioning of **K1385** (−12.8 kcal/mol; Figure 3E,F) to **K1383** and **K1384** was proposed in the *h*AChE active site. Accordingly, the 6-Cl-THA unit is oriented by parallel π-π/cation-π stacking to Tyr72 (4.0 Å) and disordered π-π bonding to Trp286 towards the outside of the cavity, suggesting its anchoring in the PAS. Indeed, this binding pose is possible since it has been previously reported that THA itself can be a CAS as well as a PAS binder [72]. Notwithstanding the PAS orientation of 6-Cl-THA, its chlorine atom also exerted alkyl-π bonding to Tyr124 pointing out to the flexibility of this aromatic residue. This observation is in line with the findings where Tyr133, and Tyr337 in conjunction with Tyr124 regulates the flow of the substrate into the catalytic site from the entrance thus presuming high-flexibility of these residues [73]. The scutellarin moiety in **K1385**-*h*AChE complex resides centrally in CAS. Herein, scutellarin revealed a specific T-shaped π-π bonding with Phe338 and Tyr33. Trp86 is also implicated in π-π interactions to scutellarin and additionally forms a hydrogen bond with one of the methoxy groups. Ser203 is the only residue from the catalytic triad being involved in the ligand settling by van der Waals bonding.

The missing cation-π interaction together with opposite **K1385** anchoring in the *h*AChE active site are plausibly the culprits of the slightly reduced affinity to the enzyme. This is somewhat counterbalanced by not displaying unfavorable donor-donor interaction with Tyr337 like in case of **K1383**- and **K1384**-*h*AChE complexes, thus the overall discrepancy in enzyme affinity is marginal.

**Figure 4:**
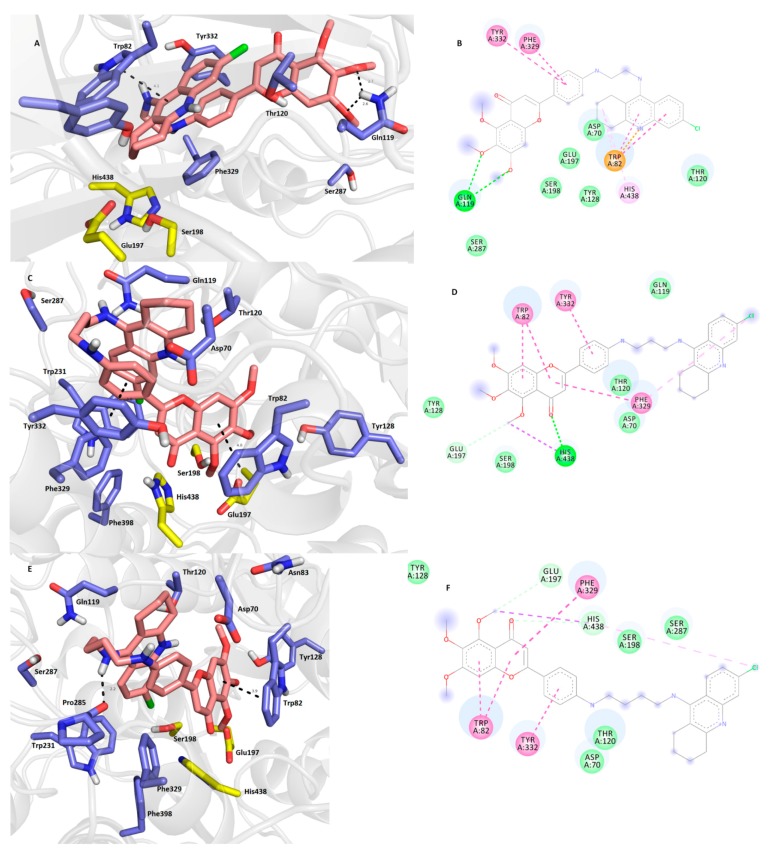
Superimposition of **K1383** (**A**,**B**), **K1384** (**C**,**D**) and **K1385** (**E**,**F**) in *h*BChE active site. Important amino acid residues involved in enzyme-ligand interaction are depicted in light-blue carbon atoms, ligands in salmon carbons and catalytic triad in yellow (**A**,**C**,**E**). Three-dimensional Figures (**A**,**C**,**E**) were created by PyMol viewer 1.3., 2D Figures (**B**,**D**,**F**) were built by BIOVIA Discovery Studio software.

Focused on the **K1383**-*h*BChE complex (−12.8 kcal/mol, Figure 4A,B), we perceived that the 6-Cl-THA heterocycle is situated in the CAS of *h*BChE facing in a parallel manner towards Trp82 (4.1 Å) via π-π/cation-π. The phenyl ring of scutellarin moiety is accommodated by typical T-shaped π-π interactions with Tyr332 (3.7 Å) and simultaneously with Phe329 (4.0 Å). Two methoxy groups from scutellarin moiety form triangular bonding to Gln119 (2.7 Å and 2.6 Å). Regarding the catalytic triad, His438 contacts the cyclohexyl moiety of tacrine core via an alkyl-π interaction while Glu197 with Ser198 were tightly bound by a non-specific van der Waals hydrophobic interaction. Contrary to *h*AChE, several important π-π interactions to scutellarin were omitted due to “the more-aliphatic nature” of *h*BChE PAS.

Opposite ligand settling can be observed for **K1384**-*h*BChE (−11.1 kcal/mol; Figure 4C,D) complex. In this case, the scutellarin moiety is preferentially oriented toward the enzyme cavity providing π-π stacking to Trp82 (4.0 Å). More importantly, all the catalytic triad residues are involved in ligand harboring; these are conventional hydrogen bonds of His438 to a ketone and π-sigma contact to a methoxy group, van der Waals interaction with Ser198 and carbon-hydrogen bonding with Glu197. 6-Cl-THA is further stabilized by its chlorine atom via a π-alkyl connection to Phe329. Interestingly, the latter amino acid residue is also involved in a π-π interactions with the scutellarin unit. Generally, such an opposite orientation of **K1384** may sufficiently explain the high affinity to *h*BChE, leading us to conclude that scutellarin itself is a more potent CAS binder than 6-Cl-THA. We note that our observation is in line with the data found from the in vitro tests.

Taking into account the most *h*AChE selective ligand under study, **K1385**, and its anchoring to *h*BChE (−11.1 kcal/mol; Figure 4E,F), several conclusions justifying its low *h*BChE inhibitory activity can be drawn. Firstly, the scutellarin moiety is accommodated into CAS like in **K1384**-*h*BChE, however, in slightly diverse manner. We found parallel π-π stacking with Trp82 (3.9 Å) and also with Phe329. In this case, Phe329 does not connect to the chlorine atom from the THA core. On the contrary, chlorine forms a π-alkyl contact with His438 from the catalytic triad. Other members of catalytic triad (Ser198, Glu197) similarly provide ligand bonding to scutellarin like in case of **K1384**-*h*BChE. More importantly, the four carbon chain between the two moieties is presumably too long to efficiently contact CAS and PAS concomitantly, thus causing the 6-Cl-THA moiety to jut out of from the active site.

Our computational studies confirmed that the relationship between *h*BChE and derivatives from the 6-Cl-THA-scutellarin subset involves a lower affinity to the *h*BChE active site. This finding, supported by in vitro data, can be rationalized by the primary structure of both cholinesterases where six of these residues in *h*BChE are changed to smaller ones, including Tyr 72 to Asn 68, Tyr 124 to Gln 119, Trp 286 to Ala 277, Phe 295 to Leu286, Phe 297 to Val 288, and Tyr 337 to Ala 328 [74]. These smaller residues significantly enlarge the entrance radius, which makes it possible for bulkier substrates to enter the active site.

## 3. Materials and Methods

### 3.1. General Information

All reagents were of reagent grade quality obtained from Sigma-Aldrich (Prague, Czech Republic). Solvents for synthesis were obtained from Penta Chemicals Co. (Chrudim, Czech Republic). All experiments were carried out under nitrogen atmospheres. Thin layer chromatography (TLC) was performed on aluminium sheets precoated with silica gel 60 F254 (Merck, Prague, Czech Republic) and then visualized by UV 254 nm and 366 nm. Column chromatography was performed at normal pressure on silica gel 100 (particle size 0.063–0.200 mm, 70–230 mesh ASTM, Fluka, Prague, Czech Republic). Mass spectra were recorded using a combination of high performance liquid chromatography (HPLC) and mass spectrometry (MS). The Dionex Ultimate 3000 LC-MS analytical system was connected with an Orbitrap Q Exactive Plus hybrid spectrometer (Thermo Fisher Scientific, Bremen, Germany). ^1^H-NMR and ^13^C-NMR spectra were recorded with a Varian Mercury VX BB 300 (operating at 300 MHz for ^1^H and 75 MHz for ^13^C) or on a Varian S500 spectrometer operating at 500 MHz for ^1^H and 126 MHz for ^13^C (Varian Inc. Palo Alto, CA, USA). Chemical shifts are reported in parts per million (ppm, δ) relative to TMS. The assignment of chemical shifts is based in standard NMR experiments (^1^H, ^13^C, ^1^H-^1^H COSY, ^1^H-^13^C HSQC, HMBC, DEPT). Melting points were measured using a M-565 automated melting point recorder (Büchi, Flawil, Switzerland).

### 3.2. 3,4,5-Trimethoxyphenyl Acetate *(**4**)*

A mixture of 3,4,5-trimethoxyphenol (**3**, 27 mmol) and sodium acetate (61 mmol) in acetic anhydride (265 mmol) was heated at 110 °C for 2 h. The mixture was concentrated under vacuum, diluted with water (50 mL), and extracted with dichloromethane (50 mL). The organic layer was washed with water (50 mL), and dried over anhydrous sodium sulphate to afford product **4** as a light brown oil, which solidified and was further crystallized from ethanol. Yield: 98%. Spectral data were in good agreement with literature data [43].

### 3.3. 1-(6-Hydroxy-2,3,4-trimethoxyphenyl)ethane-1-one *(**5**)*

Boron trifluoride-diethyl ether complex (BF_3_·Et_2_O, 17.9 mmol) was added dropwise to a solution of **4** (5.08 mmol) in glacial acetic acid (1.5 mL). The mixture was heated at 70 °C during 2 h, then poured into 10% aqueous sodium hydroxide (30 mL). After washing with ether, the aqueous layer was cooled, acidified wit conc. HCl, and extracted with dichloromethane (50 mL). The combined organic layers were dried oved sodium sulphate to obtain the product as a brown oil [43]. Yield: 76%. ^1^H-NMR (CDCl_3_) δ 13.43 (s, 1H), 6.23 (s, 1H), 3.99 (s, 3H), 3.88 (s, 3H), 3.78 (s, 3H), 2.65 (s, 3H); ^13^C-NMR (CDCl_3_) δ 203.3, 161.8, 160.0, 155.2, 134.7, 108.4, 96.1, 60.9 (2 *×* C), 56.0, 31.9.

### 3.4. (E)-3-(4-Bromophenyl)-1-(6-hydroxy-2,3,4-trimethoxyphenyl)-prop-2-en-1-one *(**6**)*

To a stirred solution of **5** (13 mmol) and 4-bromobenzaldehyde (14 mmol) in ethanol (30 mL) was added 30% aqueous potassium hydroxide solution (45 mmol). The reaction mixture was stirred for 72 h at room temperature and quenched with ice-cold water (50 mL), acidified with 10% HCl, filtered and washed with water (20 mL). The crude product was recrystallized with ethanol to afford the product as yellow oil [44]. Yield: 85%. ^1^H-NMR (CDCl_3_) δ 13.60 (s, 1H), 7.93 (d, *J* = 15.7 Hz, 1H), 7.74 (d, *J* = 15.7 Hz, 1H), 7.55 (d, *J* = 8.3 Hz, 2H), 7.50 (d, *J* = 8.3 Hz, 2H), 6.30 (s, 1H), 3.93 (s, 3H), 3.91 (s, 3H), 3.84 (s, 3H); ^13^C-NMR (CDCl_3_) δ 198.9, 169.0, 166.7, 161.2, 147.9, 141.6, 140.6, 138.7, 138.5, 137.3, 136.0, 133.5, 130.8, 114.9, 102.9, 68.2, 67.6, 62.4.

### 3.5. 2-(4-Bromophenyl)-5,6,7-trimethoxy-4H-chromen-4-one *(**7**)*

Compound **6** (1 mmol) was dissolved in DMSO (4 mL). Iodine (1 mmol) was added to the reaction mixture and refluxed at 170 °C for 3 h. The reaction mixture was quenched with ice-cold water (20 mL) and the precipitate was filtered and washed with saturated sodium thiosulfate (10 mL). The resulting product was purified by column chromatography using a mobile phase heptane: ethyl acetate (3:1) to obtain desired intermediate as yellow solid [44]. Yield: 20%. m.p.: 82.1–84.6 °C. ^1^H-NMR (DMSO-*d*_6_) δ 7.98 (m, 2H), 7.74 (m, 2H), 7.20 (s, 1H), 6.83 (s, 1H), 3.93 (s, 3H), 3.79 (s, 3H), 3.75 (s, 3H); ^13^C-NMR (DMSO-*d*_6_) δ 176.1, 159.5, 158.1, 154.4, 151.9, 140.3, 132.6, 132.5, 130.6, 128.7, 128.4, 125.6, 112.6, 108.3, 97.8, 62.3, 61.5, 56.9.

### 3.6. 6,9-Dichloro-1,2,3,4-tetrahydroacridine *(**8**)*

To a mixture of 4-chloroanthranilic acid (54 mmol) and cyclohexanone (52 mmol) was carefully added phosphorus oxychloride (332 mmol) in ice bath. The resulting mixture was heated under reflux for 2 h. The mixture was poured into a mixture of crashed ice (200 mL), chloroform (50 mL) and 25% solution of ammonia. The organic layer was washed with water (50 mL), dried over anhydrous CaCl_2_ and concentrated in vacuo. The mixture was purified by column chromatography using mobile phase ethyl acetate/petroleum-ether (1:6). Silica gel for column chromatography was washed by triethylamine before starting purification. Intermediate **8** was obtained as brown solid. Yield: 70%. Spectral data were in good agreement with the literature characterization [46].

### 3.7. Procedure for the Preparation of N-(6-Chloro-1,2,3,4-tetrahydrocridin-9-yl)alkane-1,ω-diamines ***9**–**15***

The reaction mixture of phenol (18 mmol) and 6,9-dichloro-1,2,3,4-tetrahydroacridine (**8**, 2.01 mmol) was heated to 90 °C to form a homogenous reaction solution. To this mixture appropriate 1,ω-diaminoalkane (12 mmol) was added and the reaction mixture was increased to 130 °C. After 4 h, the mixture was cooled to the room temperature and 2 M aqueous solution of sodium hydroxide (50 mL) was added. The solution was extracted with dichloromethane (100 mL). The organic layer was washed with brine (50 mL) and water (50 mL) and dried with sodium sulphate. The appropriate intermediate was purified by column chromatography using an ethyl acetate/metanol/triethylamine (8:1:0.2) mobile phase. Yield: 70–95%. Spectral data were in good agreement with the literature characterization [23].

### 3.8. General Procedure for the Preparation of 6-Chlorotacrine-scutellarin Hybrids ***K1383**–**K1389***

To the appropriate *N*-(6-chloro-1,2,3,4-tetrahydroacridin-9-yl)alkane-1-ω-diamine **9**–**15** (0.3 mmol) dissolved in dry toluene under an inert atmosphere compound **7** (0.26 mmol), palladium complex (0.002 mmol), consisting of Pd_2_(dba)_3_ (tri(dibenzylideneacetone)dipalladium (0)), BINAP (2,2′-bis(diphenylphosphino)-1,1′-binaphthyl) and NaO*t*-Bu, were added [47]. The reaction mixture was held at 110 °C for 24 h. The solvent was evaporated under the reduced pressure and the residue purified by column chromatography using chloroform:methanol (50:1) as a mobile phase. The final products **K1383**–**K1389** were obtained as yellow solids. Yield: 15–88%.

*2-(4-(2-Chloro-1,2,3,4-tetrahydroacridin-9-ylamino)ethylamino)phenyl-5,6,7-trimethoxy-4H-chromen-4-one* (**K1383**). m.p.: 123.7–128.1 °C. Yield: 40%. ^1^H-NMR (CDCl_3_) δ 7.96 (m, 2H), 7.67 (d, *J* = 8.8 Hz, 2H), 7.23 (dd, *J* = 9.0, 2.1 Hz, 1H), 6.78 (s, 1H), 6.71 (d, *J* = 8.8 Hz, 2H), 6.49 (s, 1H), 3.98 (s, 3H), 3.98 (s, 3H), 3.92 (s, 3H), 3.87 (t, *J* = 5.4 Hz, 2H), 3.54 (m, 2H), 3.04 (m, 2H), 2.67 (m, 2H), 1.85 (m, 4H); ^13^C-NMR (CDCl_3_) δ 177.2, 161.7, 157.5, 154.4, 152.4, 151.4, 150.5, 140.2, 135.2, 128.2, 127.6 (2 *×* C), 125.0, 124.5, 120.1, 117.7, 115.8, 112.7, 112.7 (2 *×* C), 150.6, 96.2, 62.2, 61.5, 56.3, 47.6, 44.1, 29.7, 24.7, 22.6, 22.0, ESI-HRMS: *m*/*z* 293.6068 [M + 2H]^+^ (calculated for: [C_33_H_33_ClN_3_O_5_]^+^/2 293.6000).

*2-(4-(2-Chloro-1,2,3,4-tetrahydroacridin-9-ylamino)propylamino)phenyl-5,6,7-trimethoxy-4H-chromen-4-one* (**K1384**). m.p.: 103.6–106.3 °C. Yield: 15%. ^1^H-NMR (CDCl_3_) δ 8.00 (d, *J* = 9.1 Hz, 1H), 7.94 (d, *J* = 2.1 Hz, 1H), 7.66 (d, *J* = 8.5 Hz, 2H), 7.17 (m, 2H), 6.77 (s, 1H), 6.73 (d, *J* = 8.6 Hz, 2H), 6.48 (s, 1H), 3.98 (s, 3H), 3.98 (s, 3H), 3.92 (s, 3H), 3.87 (t, *J* = 6.4 Hz, 2H), 3.43 (m, 2H), 3.02 (d, *J* = 6.0 Hz, 2H), 2.62 (d, *J* = 5.4 Hz, 2H), 2.12 (d, *J* = 6.4 Hz, 2H), 1.85 (m, 4H); ^13^C-NMR (CDCl_3_) δ 177.2, 161.9, 157.4, 154.4, 152.4, 150.7, 140.2, 135.8, 128.9, 128.7, 128.2, 127.5 (2 *×* C), 125.3, 125.2, 124.9, 119.5, 112.7, 112.5 (2 *×* C), 105.4, 96.2, 62.2, 61.5, 56.3, 46.9, 41.1, 30.1, 29.7, 24.5, 22.4, 21.6, ESI-HRMS: *m*/*z* 300.6164 [M + 2H]^+^ (calculated for: [C_34_H_35_ClN_3_O_5_]^+^/2 300.6079).

*2-(4-(2-Chloro-1,2,3,4-tetrahydroacridin-9-ylamino)butylamino)phenyl-5,6,7-trimethoxy-4H-chromen-4-one* (**K1385**). m.p.: 102.1–104.5 °C. Yield: 64%. ^1^H-NMR (CDCl_3_) δ 7.98 (d, *J* = 9.1 Hz, 1H), 7.91 (d, *J* = 2.2 Hz, 1H), 7.61 (m, 2H), 7.19 (dd, *J* = 9.1, 2.2 Hz, 1H), 6.77 (s, 1H), 6.65 (m, 2H), 6.47 (s, 1H), 3.98 (s, 3H), 3.96 (s, 3H), 3.90 (s, 3H), 3.68 (t, *J* = 7.0 Hz, 2H), 3.24 (t, *J* = 6.6 Hz, 2H), 3.03 (m, 2H), 2.62 (m, 2H), 1.85 (m, 6H), 1.77 (m, 2H); ^13^C-NMR (CDCl_3_) δ 177.2, 161.9, 157.4, 156.6, 154.4, 152.4, 152.2, 150.8, 143.0, 140.1, 135.5, 131.2, 130.9, 127.5, 125.0, 124.7, 124.0, 119.0, 116.8, 114.0, 112.7, 112.2, 105.2, 96.2, 62.1, 61.5, 56.2, 42.8, 31.6, 29.6, 28.9, 26.3, 24.6, 22.5, 21.7, ESI-HRMS: *m*/*z* 307.6243 [M + 2H]^+^ (calculated for: [C_35_H_37_ClN_3_O_5_]/2^+^ 307.6157).

*2-(4-(2-Chloro-1,2,3,4-tetrahydroacridin-9-ylamino)pentylamino)phenyl-5,6,7-trimethoxy-4H-chromen-4-one* (**K1386**). m.p.: 84.9–87.5 °C. Yield: 88%. ^1^H-NMR (CDCl_3_) δ 7.97 (m, 2H), 7.64 (m, 2H), 7.22 (dd, *J* = 9.1, 2.2 Hz, 1H), 6.76 (s, 1H), 6.62 (m, 2H), 6.47 (s, 1H), 3.97 (s, 3H), 3.96 (s, 3H), 3.90 (s, 3H), 3.64 (t, *J* = 7.1 Hz, 2H), 3.19 (t, *J* = 6.8 Hz, 2H), 3.04 (m, 2H), 2.65 (m, 2H), 1.86 (m, 4H), 1.78 (m, 2H), 1.70 (m, 2H), 1.55 (m, 2H); ^13^C-NMR (CDCl_3_) δ 177.3, 161.9, 157.4, 154.4, 152.5, 152.3, 150.9, 140.1, 139.3, 135.5, 127.5 (2 *×* C), 125.1, 124.8, 119.0, 116.9, 114.0, 112.8, 112.2 (2 *×* C), 105.2, 96.2, 62.2, 61.5, 58.4, 56.3, 50.7, 48.8, 43.0, 31.9, 31.2, 29.7 (2 *×* C), 28.8; ESI-HRMS: *m*/*z* 314.6320 [M + 2H]^+^ (calculated for: [C_36_H_39_ClN_3_O_5_]^+^/2 314.6235).

*2-(4-(2-Chloro-1,2,3,4-tetrahydroacridin-9-ylamino)hexylamino)phenyl-5,6,7-trimethoxy-4H-chromen-4-one* (**K1387**). m.p.: 74.9–77.3 °C. Yield: 67%. ^1^H-NMR (CDCl_3_) δ 8.02 (m, 2H), 7.68 (m, 2H), 7.24 (dd, *J* = 9.1, 2.1 Hz, 1H), 6.77 (s, 1H), 6.64 (m, 2H), 6.49 (s, 1H), 3.98 (s, 3H), 3.97 (s, 3H), 3.91 (s, 3H), 3.68 (t, *J* = 7.2 Hz, 2H), 3.18 (t, *J* = 6.9 Hz, 2H), 3.07 (m, 2H), 2.66 (m, 2H), 1.88 (m, 4H), 1.76 (m, 2H), 1.68 (m, 2H), 1.48 (m, 4H); ^13^C-NMR (CDCl_3_) δ 177.3, 162.0, 157.4, 154.4, 152.5, 151.0, 140.1, 139.3, 127.5 (2 *×* C), 125.2, 124.8, 124.5, 123.9, 123.4, 119.0, 114.0, 112.8, 112.2 (2 *×* C), 105.2, 96.2, 62.2, 61.5, 56.3, 48.7, 43.2, 33.8, 31.9, 31.6, 31.4 (2 *×* C), 26.7, 26.5, 24.5; ESI-HRMS: *m*/*z* 321.6399 [M + 2H]^+^ (calculated for: [C_37_H_41_ClN_3_O_5_]^+^/2 321.6314).

*2-(4-(2-Chloro-1,2,3,4-tetrahydroacridin-9-ylamino)heptylamino)phenyl-5,6,7-trimethoxy-4H-chromen-4-one* (**K1388**). m.p.: 82.4–86.8 °C. Yield: 60%. ^1^H-NMR (CDCl_3_) δ 7.98 (m, 2H), 7.67 (d, *J* = 8.8 Hz, 2H), 7.25 (dd, *J* = 9.0, 2.2 Hz, 1H), 6.77 (s, 1H), 6.63 (m, 2H), 6.49 (s, 1H), 3.98 (s, 3H), 3.97 (s, 3H), 3.91 (s, 3H), 3.61 (t, *J* = 7.3 Hz, 2H), 3.16 (t, *J* = 7.1 Hz, 2H), 3.05 (m, 2H), 2.64 (m, 2H), 1.86 (m, 4H), 1.74 (m, 2H), 1.62 (m, 2H), 1.43 (m, 6H); ^13^C-NMR (CDCl_3_) δ 177.3, 162.0, 157.4, 154.4, 152.5, 152.2, 151.0, 140.1, 136.7, 135.4, 127.5 (2 *×* C), 125.1, 124.7, 119.0, 117.1, 114.2, 112.8, 112.2 (2 *×* C), 105.2, 96.2, 62.2, 61.5, 56.3, 49.1, 43.3, 31.5, 29.7, 29.2, 29.0, 26.9, 26.7, 24.4, 22.6, 21.9; ESI-HRMS: *m*/*z* 328.6477 [M + 2H]^+^ (calculated for: [C_38_H_43_ClN_3_O_5_]^+^/2 328.6392).

*2-(4-(2-Chloro-1,2,3,4-tetrahydroacridin-9-ylamino)octylamino)phenyl-5,6,7-trimethoxy-4H-chromen-4-one* (**K1389**). m.p.: 69.7–73.7 °C. Yield: 29%. ^1^H-NMR (CDCl_3_) δ 7.99 (m, 2H), 7.68 (d, *J* = 8.8 Hz, 2H), 7.25 (dd, *J* = 9.1, 2.2 Hz, 1H), 6.77 (s, 1H), 6.62 (m, 2H), 6.50 (s, 1H), 3.98 (s, 3H), 3.97 (s, 3H), 3.91 (s, 3H), 3.62 (m, 2H), 3.16 (t, *J* = 7.1 Hz, 2H), 3.05 (m, 2H), 2.65 (m, 2H), 1.87 (m, 6H), 1.65 (m, 4H), 1.35 (m, 6H); ^13^C-NMR (CDCl_3_) δ 177.3, 162.0, 157.3, 154.4, 152.5, 151.0, 140.1, 135.2, 129.8, 127.9, 127.5 (2 *×* C), 125.1, 124.7, 118.9, 116.8, 113.8, 112.8, 112.2 (2 *×* C), 105.2, 96.2, 62.2, 61.5, 56.3, 49.0, 43.4, 31.9, 31.5, 29.7, 29.2 (2 *×* C), 26.9, 26.7, 24.4, 22.6, 21.9; ESI-HRMS: *m*/*z* 335.6555 [M + 2H]^+^ (calculated for: [C_39_H_45_ClN_3_O_5_]^+^/2 335.6470).

### 3.9. Biological Evaluation

#### 3.9.1. Inhibition of Human AChE and BChE

The AChE and BChE inhibitory activities of the tested compounds were determined using a modified Ellman’s method [48,49] and were expressed as IC_50_ values, i.e., the concentration of compound required for 50% reduction in cholinesterase activity. Human erythrocyte hemolyzate, prepared from blood samples, was applied as a source of AChE (EC 3.1.1.7). The blood samples were collected from healthy volunteers from the vein into a disposable syringe containing 3.8% sodium citrate solution (the ratio/citrate was 1:10 *w*/*w*). The citrate blood was centrifuged for 20 min at 2856× *g* and the plasma was removed as supernatant. The erythrocytes were washed three times with phosphate buffer (PB; 0.1 M, pH 7.4) and then hemolyzed in PB (0.01 M, Ph 7.4) in ration 1:10 (*w*/*w*), frozen and kept under −80 °C before use. Human plasma BChE (EC 3.1.1.8), 5,5′-dithiobis(2-nitrobenzoic acid) (Ellman’s reagent, DTNB), phosphate buffer solution (PBS, pH 7.4), acetylthiocholine iodide (ATCh), and butyrylthiocholine (BTCh) were purchased from Sigma-Aldrich. Polystyrene Nunc 96-well microplates with flat bottom shape (ThermoFisher Scientific, Bremen, Germany) were used for the measuring purposes. All of the assays were carried out in 0.1 M KH_2_PO_4_/K_2_HPO_4_ buffer, pH 7.4. Enzyme solutions were prepared in 1 mL aliquot with activity of 12 µkat. The assay medium (100 μL) consisted of 40 μL of 0.1 M PBS (pH = 7.4), 20 μL of 0.01 M DTNB, 10 μL of cholinesterase, 10 µL of tested compound and 20 μL of 0.01 M substrate (ATCh or BTCh iodide solution). Assayed solutions of target compounds (10 μL, 10*^−^*^3^–10*^−^*^9^ M) were preincubated with enzyme for 5 min. The reaction was initiated by addition of 20 μL of substrate. The activity was determined by measuring of the increase in absorbance at 412 nm at 37 °C in 2 min intervals using a Synergy 2 multimode microplate reader (BioTek Instruments, Winooski, VT, USA). Each concentration was assayed in triplicate. Percentage of inhibition (*I*) was calculated from the measured data as follows Equation (1):

(1) $$I=\left(1-\frac{\Delta A_{i}}{\Delta A_{0}}\right)\times 100\;[\%]$$

Δ*A*_i_ indicates absorbance change provided by cholinesterase exposed to tested compound. Δ*A*_0_ indicates absorbance change caused by intact cholinesterase, where phosphate bufffer was applied in the same was as the anticholinesterase compound. Software Microsoft Excel (Redmond, WA, USA) and GraphPad Prism version 5.02 for Windows (GraphPad Software, San Diego, CA, USA) (www.graphpad.com) were used for the statistical data evaluation. The IC_50_ values are expressed as a mean ± SEM.

#### 3.9.2. Kinetic Study of AChE Inhibition

The kinetic study of AChE inhibition was performed using Ellman’s method [48]. Following concentrations of substrate were used for the measurements: 0.156, 0.313, 0.625 and 1.250 mM. The type of inhibition and corresponding inhibition constant (*K*_i_ and *K*_i_′) were determined by the nonlinear regression analysis of the substrate velocity curves at different concentrations of tested compounds. Results for each type model of inhibition (competitive, uncompetitive, mixed and mixed-non-competitive) were compared with sum-of-squares F-test. Lineweaver-Burk (double-reciprocal) plot, used for visualization of the enzyme-inhibitor interactions, was calculated using weighted least-squares regression. All calculations were performed using the GraphPad Prism software.

#### 3.9.3. Evaluation of Antioxidant Activity

Diphenyl-1-picrylhydrazyl stable free radical assay (DPPH) [52] is a simple method to determine antioxidant activity and is expressed as EC_50_, that is the concentration of compound that causes a 50% decrease in the DPPH activity. DPPH, methanol, and Trolox (as reference standard) were purchased from Sigma-Aldrich. Polystyrene Nunc 96-well microplates with flat bottom shape (Thermo Fisher Scientific) were used for measuring purposes. All of the assays were carried out in methanol. DPPH solution was prepared at 0.2 mM concentration. The assay medium (200 µL) consisted of 100 µL of DPPH solution and 100 µL of tested compound (10*^−^*^3^–10*^−^*^6^ M). The reaction time constituted 30 min. The antioxidant activity was determined by measuring the increase in absorbance at 517 nm at laboratory temperature using Synergy 2 multimode microplate reader [75]. Each concentration was tested in triplicate. The software GraphPad Prism version 5 for Windows was used for statistical data evaluation.

#### 3.9.4. Determination of in vitro Blood-Brain Barrier Permeation

The parallel artificial membrane permeation assay (PAMPA) has been used based on reported protocol [58,59]. The filter membrane of the donor plate was coated with PBL (Polar Brain Lipid, Avanti, Alabaster, AL, USA) in dodecane (4 μL of 20 mg/mL PBL in dodecane) and the acceptor well was filled with 300 μL of PBS pH 7.4 buffer (*V*_D_). Tested compounds were dissolved first in DMSO and then diluted with PBS pH 7.4 to reach the final concentration 100 μM in the donor well. The concentration of DMSO did not exceed 0.5% (*v*/*v*) in the donor solution. 300 μL of the donor solution was added to the donor wells (*V*_A_) and the donor filter plate was carefully put on the acceptor plate so that coated membrane was “in touch” with both donor solution and acceptor buffer. Test compound diffused from the donor well through the lipid membrane (Area = 0.28 cm^2^) to the acceptor well. The concentration of the drug in both donor and the acceptor wells was assessed after 3, 4, 5 and 6 h of incubation in quadruplicate using the Synergy HT UV plate reader at the maximum absorption wavelength of each compound. Concentration of the compounds was calculated from the standard curve and expressed as the permeability (*P*e) according to Equation (2) [76,77]:

(2) $$\begin{array}{c} \log P_{e}=\log\;\left\{C\times -ln\left(1-\frac{{\left[drug\right]}_{acceptor}}{{\left[drug\right]}_{equilibrium}}\right)\right\}\;where\;C\\ \;\;\;\;\;\;=\;\left(\frac{V_{D}\times V_{A}}{\left(V_{D}+V_{A}\right)\times Area\times time}\right) \end{array}$$

#### 3.9.5. Determination of Hepatotoxicity on HepG2 Cells

Hepatotoxicity of tested compounds was evaluated using the HepG2 cell line originally from human liver hepatocellular carcinoma (ATCC, Manassas, VA, USA). These cells were plated at density 17 *×* 10^3^ per well in a 96-well plate in Dulbecco’s Modified Eagle’s Medium (DMEM; Gibco, Langley, OK, USA) with 10% FBS (Gibco) and were left to attach overnight. The incubation was performed under condition 37 °C, 5% CO_2_ and 80–95% air humidity. Stock solutons of tested compounds were prepared in DMSO (Sigma Aldrich, Prague, Czech Republic) and then diluted in DMEM medium. The final concentration of DMSO was less than 0.25%. The cell viability was detected using (3-4,5-dimethylthiazol-2-yl)-2,5-diphenyltetrazolium bromide (MTT; Sigma Aldrich) assay after 24 h of incubation with compounds [56]. Subsequently, the medium was aspirated and 100 μL MTT solution (0.5 mg/mL) in serum-free DMEM medium was added to each well. Cells were then incubated for 1 h. The medium was then discarded and purple crystals of MTT formazan were dissolved in 100 μL DMSO under shaking. The absorbance was measured with a microplate reader (BioTek) at a minimum wavelength of 570 nm. The IC_50_ values were calculated using four parametric nonlinear regression with statistic software GraphPad Prism. Data were obtained from three independent experiments performed in triplicates.

#### 3.9.6. Molecular Modeling Studies

Two structures of *h*AChE and *h*BChE were obtained from the RCSB Protein Data Bank—PDB ID: 4EY7 (crystal structure of *h*AChE) and 4BDS (crystal structure of *h*BChE) [63,64]. All receptor structures were prepared by DockPrep function of UCSF Chimera (version 1.4) and converted to pdbqt-files by AutodockTools (v. 1.5.6) [78,79]. Flexible residues selection was based on previous experience (*h*AChE) or spherical region around the binding cavity [80,81,82,83]. Three-dimensional structures of ligands were built by Open Babel (v. 2.3.1), minimized by Avogadro (v 1.1.0) and converted to pdbqt-file format by AutodockTools [84]. The docking calculations were done by Autodock Vina (v. 1.1.2) with the exhaustiveness of 8 [85]. Calculation was repeated ten times for each ligand and receptor and the best-scored result was selected for manual inspection. The visualization of enzyme-ligand interactions was prepared using 1.3. (The PyMOL Molecular Graphics System, Version 1.5.0.4 Schrödinger, LLC, Mannheim, Germany). 2D diagrams were created with Discovery Studio 2016 Client.

## 4. Conclusions

While the exact cause(s) of Alzheimer’s disease remain unknown, new therapies aimed at a number of biological targets have been identified to design new therapies. In this context, we report the design, synthesis and pharmacological profiles of some novel multi-functional anti-AD agents **K1383**–**K1389** containing 6-Cl-THA and scutellarin fragments. These derivatives were proven to be potent ChE inhibitors exerting selective AChE dual binding site character. Several of novel hybrids **K1383**–**K1385** were better *h*AChE inhibitors in relation to a reference standard, 6-Cl-THA. The most prominent hybrid **K1383** was found to be a very potent *h*AChE inhibitor (IC_50_ = 1.63 nM). The AChE kinetic analysis revealed a mixed, non-competitive mode of AChE inhibition. Although designed as potential antioxidants, the free scavenging ability was lost (EC_50_ higher than 10^−4^ M) by the hybridization. A probable reason is the presence of methoxy groups in the scutellarin moiety instead of free phenolic groups which could presumably confer antioxidant potential. An important issue that needs to be addressed in the development of novel tacrine-based MTDLs is their potential hepatotoxicity. Our preliminary data verified on HepG2 cell line suggested a comparable hepatotoxic profile of **K1383**–**K1389** as the standard 6-Cl-THA. Moreover, the PAMPA assay predicted a probability of some hybrids, namely **K1384**, **K1388**, **K1389**, to cross the BBB while the remainder demonstrated rather uncertain CNS permeation via a passive diffusion model. This work presents a novel tacrine-based family as potential anti-AD agents, but further in vitro and in vivo testing are needed to fully established their real perspectives.

## Figures and Tables

**Table 1:** IC_50_ values for AChE, BChE and SI for AChE of hybrids **K1383**–**K1389**, 6-Cl-THA, **1** and **2**.

Compound	*n*	AChE IC_50_ ± SEM ^a^ (nM)	BChE IC_50_ ± SEM ^a^ (nM)	SI (AChE IC_50_/BChE IC_50_)
**1**	-	570 ± 20 ^b^	22600 ± 220 ^c^	39.7
**2**	-	32.5 ± 0.25 ^f^ (47.2 ± 0.19 ^d^)	968.9 ± 8.26 ^e^	20.5
**K1383**	2	1.63 ± 0.13 ^f^	1210 ± 70 ^g^	742
**K1384**	3	1.90 ± 0.10 ^f^	174 ± 7 ^g^	92
**K1385**	4	5.15 ± 0.18 ^f^	8690 ± 40 ^g^	1687
**K1386**	5	26.7 ± 1.0 ^f^	934 ± 45 ^g^	35
**K1387**	6	31.0 ± 1.5 ^f^	6460 ± 300 ^g^	208
**K1388**	7	24.1 ± 0.9 ^f^	2810 ±110 ^g^	117
**K1389**	8	22.3 ± 0.7 ^f^	2210 ± 130 ^g^	99
**6-Cl-THA**	-	20 ± 1 ^f^	1780 ± 97 ^g^	100.68

^a^ Results are expressed as the mean of three independent measurement ± standard error of the mean (SEM); ^b^ AChE isolated from rat cortex homogenate; ^c^ BChE from rat serum; ^d^ *Electric eel* AChE activity; ^e^ BChE from equine serum; ^f^ AChE from human erythrocytes; ^g^ BChE from human plasma.

**Table 2:** Antioxidant power of hybrids **K1383**–**K1389**, 6-Cl-THA, **1**, **2** and trolox.

Compound	DPPH EC_50_ [μM]
**1**	1.3 ± 0.02 ^a^
**2**	0.47 ± 0.03 ^a^
**K1383**	>500 ^b^
**K1384**	>500 ^b^
**K1385**	>500 ^b^
**K1386**	>500 ^b^
**K1387**	>500 ^b^
**K1388**	>500 ^b^
**K1389**	>500 ^b^
6-Cl-THA	>100 ^b^
trolox	16.20 ± 0.42 ^b^

^a^ Results are expressed as µM of Trolox/µM of tested compound as the mean of at least three experiments determined by the Oxygen Radicals Absorbance Capacity by Fluorescence (ORAC-FL) [55] from reference [31] for compound **1** and from reference [42] for compound **2**; ^b^ Results are expressed as the mean of at least three experiments.

**Table 3:** Hepatotoxicity of 6-Cl-THA-scutellarin hybrids **K1383**–**K1389** and reference 6-Cl-THA.

Compound	IC_50_ HepG2 ± SEM ^a^ (μM)
**K1383**	3.40 ± 1.05
**K1384**	5.77 ± 1.68
**K1385**	3.29 ± 0.82
**K1386**	3.89 ± 1.08
**K1387**	7.76 ± 1.47
**K1388**	>32
**K1389**	>16
6-Cl-THA	7.13 ± 0.11

^a^ Values are expressed as the mean ± SEM of three independent experiments.

**Table 4:** Prediction of BBB crossing for hybrids **K1383**–**K1389** and reference compound 6-Cl-THA.

Compound	*P*_e_ (×10^−6^ cm/s) ^a^	Prediction ^b^
**K1383**	2.88 ± 0.10	CNS±
**K1384**	5.01 ± 2.20	CNS+
**K1385**	2.60 ± 0.55	CNS±
**K1386**	3.93 ± 1.00	CNS±
**K1387**	3.31 ± 0.38	CNS±
**K1388**	4.92 ± 0.02	CNS+
**K1389**	8.10 ± 3.10	CNS+
6-Cl-THA	5.00 ± 0.45	CNS+

^a^ Values are expressed as the mean ± SD of three independent experiments; ^b^ Prediction of BBB penetration by PAMPA-BBB assay; CNS+ (high BBB permeation predicted, *P*_e_ × 10^−6^ cm/s > 4.0), CNS± (BBB permeation uncertain, *P*_e_ × 10^−6^ cm/s from 4.0 to 2.0), CNS− (low BBB permeation predicted, *P*_e_ × 10^−6^ cm/s < 2.0).
